# Prevalence of chloroquine and antifolate drug resistance alleles in *Plasmodium falciparum* clinical isolates from three areas in Ghana

**DOI:** 10.12688/aasopenres.12825.2

**Published:** 2018-12-03

**Authors:** James Abugri, Felix Ansah, Kwaku P. Asante, Comfort N. Opoku, Lucas A. Amenga-Etego, Gordon A. Awandare

**Affiliations:** 1 West African Centre for Cell Biology of Infectious Pathogens, College of Basic and Applied Sciences, University of Ghana, Legon, Ghana; 2 Department of Biochemistry, Cell and Molecular Biology, College of Basic and Applied Sciences, University of Ghana, Legon, Ghana; 3 Department of Applied Chemistry and Biochemistry, Faculty of Applied Sciences, University for Development Studies, Tamale, Ghana; 4 Kintampo Health Research Centre, Ghana Health Service, Kintampo, Ghana; 5 Ledzokuku Krowor Municipal Assembly Hospital, Accra, Ghana; 6 Navrongo Health Research Centre, Navrongo, Ghana

**Keywords:** Drug resistance, Malaria, Antifolates, Chloroquine, Plasmodium falciparum

## Abstract

**Background:** The emergence and spread of resistance in *Plasmodium falciparum* to chloroquine (CQ) necessitated the change from CQ to artemisinin-based combination therapies (ACTs) as first-line drug for the management of uncomplicated malaria in Ghana in 2005. Sulphadoxine-pyrimethamine (SP) which was the second line antimalarial drug in Ghana, was now adopted for intermittent preventive treatment of malaria in pregnancy (IPTp).

**Methods: **To examine the prevalence of molecular markers associated with CQ and antifolate drug resistance in Ghana, we employed restriction fragment length polymorphism polymerase chain reaction to genotype and compare single nucleotide polymorphisms (SNPs) in the *P. falciparum* chloroquine resistance transporter ( *pfcrt,* PF3D7_0709000), multidrug resistance ( *pfmdr1, *PF3D7_0523000), bifunctional dihydrofolate reductase-thymidylate synthase ( *pfdhfr,* PF3D7_0417200) and dihydropteroate synthase ( *pfdhps,* PF3D7_0810800) genes. Parasites were collected from children with malaria reporting to hospitals in three different epidemiological areas of Ghana (Accra, Kintampo and Navrongo) in 2012-2013 and 2016-2017.

**Results: **The overall prevalence of the CQ resistance-associated *pfcrt *76T allele was 8%, whereas *pfmdr1 *86Y and 184F alleles were present in 10.2% and 65.1% of infections, respectively. The majority of the isolates harboured the antifolate resistance-associated *pfdhfr* alleles 51I (83.4%), 59R (85.9 %) and 108N (90.5%). *Pfdhps *437G and 540E were detected in 90.6% and 0.7% of infections, respectively. We observed no significant difference across the three study sites for all the polymorphisms except for *pfdhps *437G **, **which was more common in Accra compared to Kintampo for the 2016-2017 isolates. Across both *pfdhfr* and *pfdhps* genes, a large proportion (61%) of the isolates harboured the quadruple mutant combination ( **I**_51_ **R**_59_ **N**_108_/ **G**_437_).

CQ resistance alleles decreased during the 12 years after CQ withdrawal, but an mediate SP resistance alleles increased.

**Conclusion**: Surveillance of the prevalence of resistance alleles is necessary in monitoring the efficacy of antimalarial drugs.

## Introduction

Malaria remains a major global health concern especially in sub Saharan Africa. *P. falciparum* malaria is considered the most severe and also the leading cause of morbidity and mortality, especially among children under five years ( Schumacher & Spinelli, 2012). In 2016 a global estimate of 216 million malaria cases was reported, which led to about 445,000 deaths ( WHO, 2017). The global malaria mortality rate, however, has reduced by 29% since the year 2010, as a result of increased preventive and control measures ( WHO, 2016).

The use of antimalarial drugs for malaria treatment and prevention has played an integral role in the control of the disease over the decades ( Cui *et al.*, 2015; Gosling *et al.*, 2011; Greenwood, 2004; Schlitzer, 2007). Unfortunately, the emergence and the spread of drug resistant *P. falciparum* strains militated against the use of antimalarial drugs for the containment of the disease ( Lin *et al.*, 2010). *P. falciparum* chloroquine (CQ) resistant strains were first reported in the 1950s in Southeast Asia along the Cambodia–Thailand border ( Young *et al.*, 1963) and subsequently reported in other countries globally. Currently, the parasite has been reported to have developed resistance to most available artemisinin monotherapies and this is exhibited by reduced parasite clearance rates and/or treatment failures ( Dondorp *et al.*, 2009). ACTs are now the frontline drugs for treating uncomplicated *P. falciparum* malaria in almost all countries that are endemic with malaria, including Ghana ( WHO, 2016).

Point mutations in specific genes in the parasite genome are implicated in resistance to specific antimalarial drugs ( Cui *et al.*, 2015; Fidock *et al.*, 2000; Sidhu *et al.*, 2002). A point mutation in the *P. falciparum* chloroquine resistance transporter gene ( *pfcrt*, PF3D7_0709000 *)* that replaces lysine with threonine at codon 76 had become a common single nucleotide polymorphism (SNP) in parasite populations as it is a critical mediator of resistance to CQ ( Babiker *et al.*, 2001). In addition, mutations in the *P. falciparum* multidrug resistance gene 1 ( *pfmdr1*, PF3D7_0523000) that result in amino acid substitutions at positions N86Y and Y184F have been reported to confer parasite resistance to CQ, amodiaquine (AQ) and lumefantrine (L) ( Duraisingh & Cowman, 2005). These mutations are believed to interfere with heme polymerization by preventing the accumulation of active drug within the food vacuole ( Djimdé *et al.*, 2001b).

*P.falciparum* resistance to Sulfadoxine-pyrimethamine (SP) has been linked to point mutations in the bifunctional dihydrofolate reductase-thymidylate synthase ( *pfdhfr*, PF3D7_0417200) and dihydropteroate synthase ( *pfdhps*, PF3D7_0810800) genes ( Cowman *et al.*, 1988; Triglia *et al.*, 1997; Wang *et al.*, 1997). Resistance to antifolate drugs such as SP is known to be mediated by basal point mutations in these genes that result in amino acid substitutions at positions S108N and A437G in *pfdhfr* and *pfdhps* proteins respectively ( Mita *et al.*, 2014). Overall, studies have shown that additional point mutations in these drug resistant genes on top of the basal mutation makes parasites more refractory to the drug ( Mita *et al.*, 2014), and correlates with increased treatment failure ( Plowe *et al.*, 1997; Sibley *et al.*, 2001). Therefore, parasites harbouring haplotypes that include the different SNP alleles in combination have been shown to confer higher resistance to the specific drugs. In this regard, the combined quintuple mutant haplotype ( *pfdhfr* **I**_51_ **R**_59_ N _108_ + *pfdhps* **G**_437_ **E**_540_) has been correlated with high SP treatment failure in East Africa ( Kublin *et al.*, 2002; Omar *et al.*, 2001).

In Ghana, prior to the withdrawal of CQ a prevalence range of between 46%–98% of the mutant *pfcrt* 76T was reported across five sentinel sites ( Duah *et al.*, 2007). Interestingly, studies in other settings have shown that the replacement of CQ with ACTs resulted in a decline in the frequency of the mutant alleles and concomitant restoration of CQ susceptibility ( Laufer *et al.*, 2006; Mwai *et al.*, 2009). In a study that was conducted in Tanzania, more than 90% recovery of the sensitive *pfcrt* K76 allele was reported after 10 years of CQ use being officially discontinued ( Mohammed *et al.*, 2013). Follow -up studies in Ghana have reported a decline in the prevalence of *pfcrt* 76T and pfmdr1 86Y but an increasing prevalence *pfdhfr* I 51, R 59, N 108 and 437G resistant alleles from 2003 to 2010 ( Duah *et al.*, 2013; Duah *et al.*, 2012)

This study sought to ascertain the population trends in the prevalence of known drug-resistance-related point mutations in *pfcrt, pfmdr1, pfdhfr* and *pfdhps* in clinical isolates from three different malaria-endemic areas in Ghana a decade following the introduction of ACTs.

## Methods

### Ethical consideration

This study was approved by the Ethics Committees of the Ghana Health Service (GHS-ERC:12/05/12), the Kintampo Health Research Centre (KHRCIEC/FEA/2011-13), the Navrongo Health Research Centre (NHRC-IRB135/08/2012) and the Noguchi Memorial Institute for Medical Research (NMIMR) (NMIMR-IRB CPN 004/11-12). Informed consent of parents or guardians for all participants was obtained. An additional assent was also obtained from children aged 10–14 years prior to recruitment.

### Study sites and sample collection

This study leveraged the availability of samples from a concurrent study at the time on erythrocyte invasion mechanisms and whole genome sequencing of the malaria parasites. The appropriate sample collection at the time to meet the erythrocyte invasion studies, the whole genome sequencing of the malaria parasites was adopted. We used the samples so gotten to carry out the drug resistance study. The choice of 2–14 years was premised on development of immunity that was key in the erythrocyte invasion study.

Parasite isolates were obtained from children aged 2–14 years, diagnosed with malaria at Municipal hospitals in Kintampo North Municipality (here after referred as Kintampo; 2012–2013 and 2016–2017), Accra (2016–2017) and Navrongo (2012–2013), in Ghana. Kintampo is a tropical zone in the Brong Ahafo region with all year round high malaria transmission, whereas Navrongo is a savannah zone in the Upper East region where malaria transmission is seasonal and rainfall-dependent ( Owusu-Agyei *et al.*, 2009). Accra lies within the coastal savannah area with low seasonal malaria transmission ( Klinkenberg *et al.*, 2008). Malaria transmission in Accra peaks during the June to August rainy season. The entomological inoculation rate (EIR) in Kintampo, Navrongo and Accra are >250, >100 and <19.2 infectious bites per person per year, respectively ( Kasasa *et al.*, 2013; Klinkenberg *et al.*, 2008). These three regions represent the different malaria transmission intensity zones in the country (Accra<Navrongo<Kintampo), and the study participants have been characterized in greater detail in our previous reports ( Ademolue *et al.*, 2017; Mensah-Brown *et al.*, 2017). Samples were obtained from participants during the rainy seasons at the respective study sites. *P. falciparum* genomic DNA was analyzed for the prevalence of known antimalarial drug resistance SNPs in *pfcrt* (K76T), *pfmdr1* (N86Y and Y184F), *pfdhfr* (N51I, C59R and S108N) and *pfdhps* (A437G and K540E) across the three study sites. Malaria was diagnosed using the first response ®malaria Ag. (HRP2) card test (Premier Medical Corporation, Ltd., Mumbai, India) and confirmed by microscopy. Venous blood samples were obtained and depleted of leucocytes using lymphoprep gradient centrifugation, followed by passage through Plasmodipur filters (EuroProxima, Arnhem, Netherlands), and the resulting infected red blood cells were stored at -20°C until DNA extraction.

**Figure 1.**
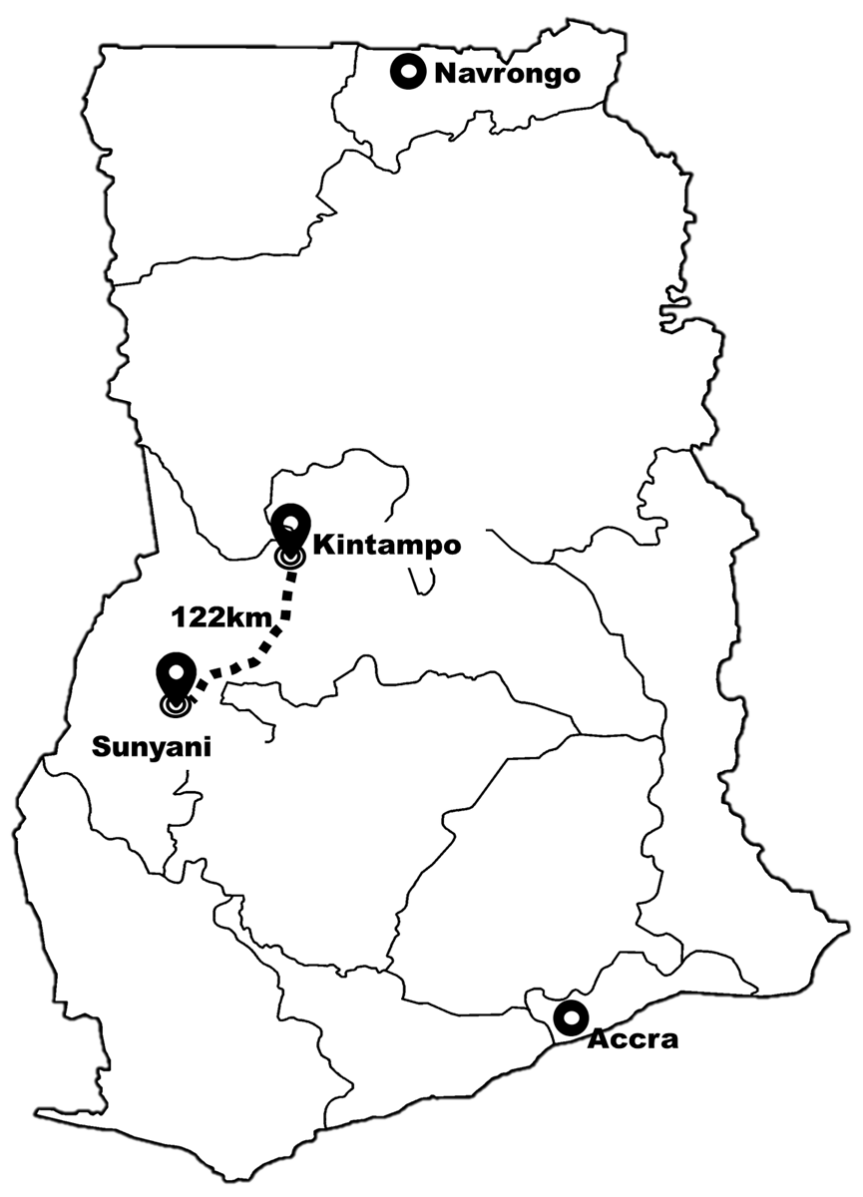
A map of Ghana showing our study sites. The distance between Sunyani and Kintampo is approximately 122 Km. Housing structures in both localities are largely similar with a slight difference in the vegetation but almost the same geospatial characteristics.

### Extraction of genomic DNA and nested PCR

*Plasmodium* gDNA was extracted from the samples using the QIAamp Blood Midi Kit (Qiagen, Manchester, UK) as per manufacturer’s instructions and stored at -20°C. Both outer and nested PCRs were carried out to amplify regions flanking known point mutations in *pfcrt* (K76T), *pfmdr1* (N86Y and Y184F), *pfdhfr (*N51I, C59R, and S108N) and *pfdhps* (A437G and K540E) that mediate antimalarial drug resistance. All PCRs were carried out at final volume of 25 µL containing 1X of Maxima Hot Start Green PCR master mix (Thermo Scientific, Waltham, MA, USA) and 250 nM of each of the forward and the reverse primers. Five microlitres of the purified *P. falciparum* gDNA was used as template in the outer PCR and 1 µL of the resulting products was used as template DNA in the nested PCR. Previously reported primer sets and cycling conditions for both the outer and the nested PCRs were used ( Djimdé *et al.*, 2001a; Duraisingh *et al.*, 1998). Prior to the restriction digest, 5 µL of the nested PCR products were resolved on 2% agarose gel stained with ethidium bromide and images were resolved using the Amersham Imager 600 (General Electric Healthcare Life Sciences, Chicago, IL, USA).

## Restriction digestion of nested PCR amplicons

The resulting nested PCR products for each of the four genes containing the SNP alleles of interest were analyzed by restriction fragment length polymorphism (RFLP). Each of the restriction digestion reactions was set at a final volume of 15 µL containing 5 µL of the nested PCR product, 1X FastDigest Green buffer and 0.3 µL of the appropriate restriction enzyme (Thermo Scientific). The restriction enzymes used, incubation temperature, incubation time as well as the expected band sizes for the wild-type and the mutant alleles of the point mutations were as reported in previous studies ( Djimdé *et al.*, 2001a; Duraisingh *et al.*, 1998). Ten microlitres of the restriction digestion fragments were resolved on 2 % agarose gel stained with ethidium bromide and the resulting image resolved with the Amersham Imager 600 (GE, USA). Purified DNA obtained from laboratory strains of *P. falciparum* (Dd2, 3D7, FCR3, K1, 7G8 and W2) were used as controls for the sensitive and resistant alleles for each gene.

### Data analysis

Data was analyzed using the Stata version 14.2 (Texas, USA), and the GraphPad Prism (Version 6.01). Analysis of contingency tables of frequency distribution of the point mutations between the study sites were analyzed by chi-square test. In addition, allele combination frequency distribution of the 2012–2013 isolates were compared to the 2016–2017 isolates utilizing the Fisher exact test for expected lower cell counts taking each marker as independent. All statistical tests were two-tailed and statistical significance was defined at P < 0.05.

## Results

### Prevalence of alleles in *P. falciparum* genes that mediate chloroquine and antifolate drug resistance

The prevalence of antimalarial drug resistance alleles in three different transmission zones were determined and compared across sites and sampling time points.

. We did not observe any significant differences in the distribution of isolates harbouring *pfcrt* K76T, *pfmdr1* N86Y or *pfmdr1* Y184F point mutations across the three transmission zones (P > 0.05 for all three SNPs), although all the three mutant alleles were found at a higher prevalence in Navrongo (2012–2013) compared to Kintampo (2012–2013 and 2017) and Accra (2016–2017) ( Table 1). The total prevalence of *pfcrt* 76T (8%) and *pfmdr1* 86Y (10.2%) mutant alleles were comparable (P = 0.39). Compared to CQ resistance-associated alleles, higher frequencies were observed in the three study sites for all the antifolate drug resistance-associated alleles, except *pfdhps* K540E ( Table 1 and Dataset 1). The frequency distribution of isolates harbouring the *pfdhfr* 51I, 59R and 108N mutant alleles were also comparable across the study sites (P > 0.05 for all the three loci). However, the distribution of *pfdhps* 437 **G** mutant allele, was significantly different across the study sites (P = 0.01). *Pfdhps* 437G was significantly higher in Accra (2016–2017) compared to Navrongo (2012–2013) (P = 0.03), Kintampo (2012–2013) (P = 0.004) and Kintampo (2016–2017) (P = 0.004). The frequency of *pfdhps* 437G in Navrongo (2012–2013), Kintampo (2012–2013) and Kintampo (2016–2017) were comparable (P > 0.05).

**Table 1.** The prevalence of *pfcrt, pfmdr1, pfdhps* and *pfdhfr* mutant allelles in *P. falciparum* isolates.

Gene	Amino acid ^a^ Position	Amino Acid ^b^	Kintampo (2012-2013) n (%)	Navrongo (2012-2013) n (%)	Accra (2016-2017) n (%)	Kintampo (2016-2017) n (%)	Total ^c^ n (%)	P-value ^d^
***Pfcrt***	K76T	K	148 (86.3)	37 (88.1)	64 (88.9)	50 (98.0)	299 (91.7)	*0.243*
		**T**	13 (13.7)	5 (11.9)	8 (11.1)	1 (2.0)	27 (8.3)	
***Pfmdr1***	N86Y	N	103 (89.6)	47 (82.5)	72 (92.3)	50 (94.3)	272 (89.8)	*0.166*
		**Y**	12 (10.4)	10 (17.5)	6 (7.7)	3 (5.7)	31 (10.2)	
	Y184F	Y	42 (34.1)	15 (27.8)	31 (38.8)	22 (37.9)	110 (34.9)	*0.574*
		**F**	81 (65.9)	39 (72.2)	49 (61.2)	36 (62.1)	205 (65.1)	
***Pfdhfr***	N51I	N	28 (20.6)	9 (16.7)	6 (8.0)	10 (18.9)	53 (16.6)	*0.124*
		**I**	108 (79.4)	46 (83.3)	69 (92.0)	43 (81.1)	266 (83.4)	
	C59R	C	12 (17.9)	5 (16.7)	12 (15.4)	3 (5.8)	32 (14.1)	*0.256*
		**R**	55 (82.1)	25 (83.3)	66 (84.6)	49 (94.2)	195 (85.9)	
	S108N	S	17 (13.0)	5 (9.1)	4 (5.1)	4 (7.5)	30 (9.5)	*0.280*
		**N**	114 (87.0)	50 (90.9)	74 (94.9)	49 (92.5)	287 (90.5)	
***Pfdhps***	A437G	A	16 (12.7)	5 (9.1)	1 (1.3)	7 (14.0)	29 (9.4)	***0.031***
		**G**	110 (87.3)	50 (90.9)	77 (98.7)	43 (86.0)	280 (90.6)	
	K540E	K	106 (99.1)	54 (98.2	78 (100)	52 (100)	290 (99.3)	-
		**E**	1 (0.9)	1 (1.8)	0 (0)	0 (0)	2 (0.7)	

^**a**^Mutated amino acid depicted in bold, ^**b**^ P-value based on Pearson chi-Square test or Exact chi-square test for categorical variables.

### Trends in the prevalence of antimalarial drug resistance markers in the study populations

**Figure 2.**
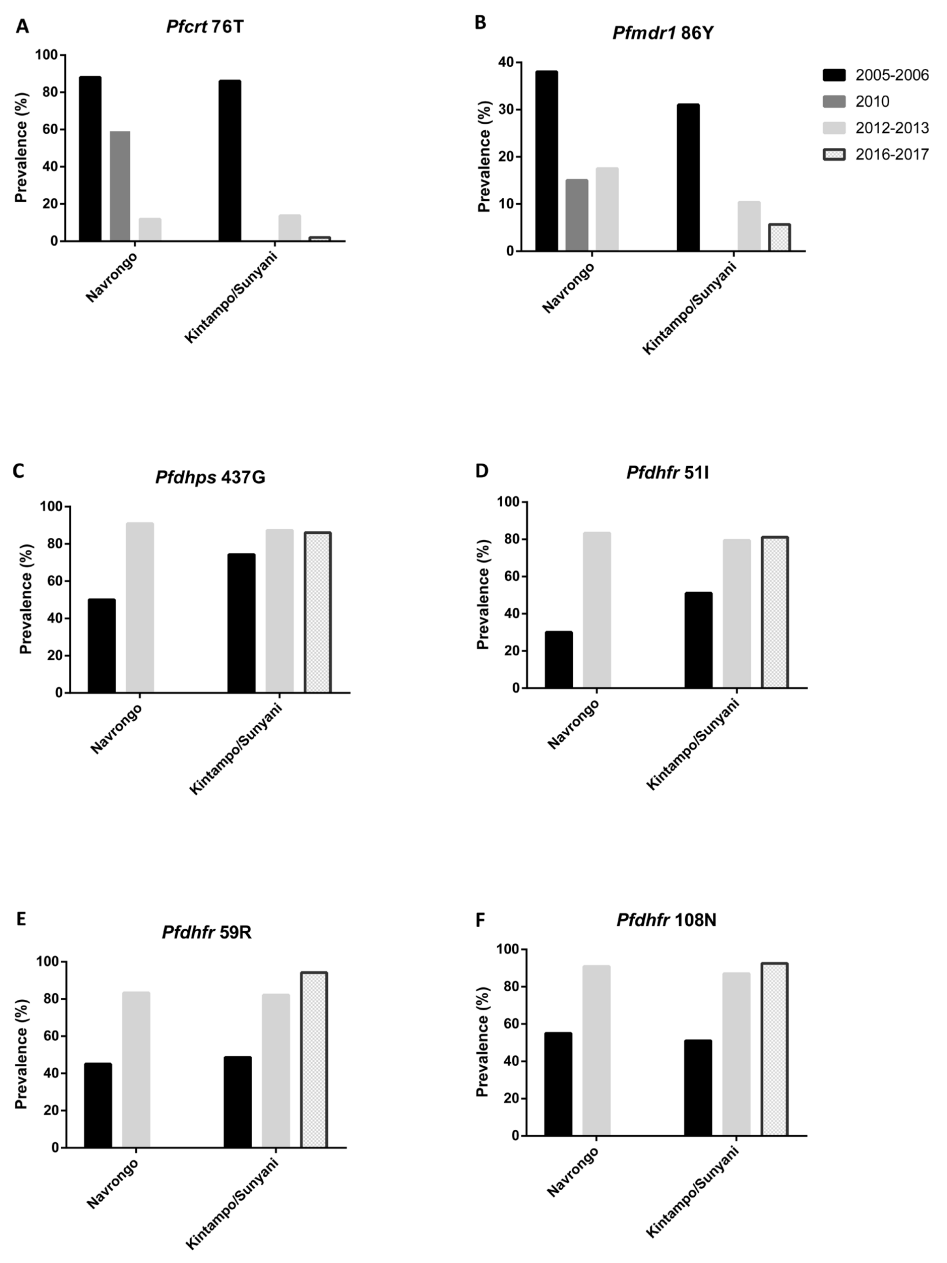
Trends in the prevalence of antimalarial drug resistant alleles from 2005 to 2017.

To investigate the dynamics of the drug resistance alleles in the selected areas, we compiled data from previous studies that reported the frequencies of the various mutations in the same or near-by communities. Thus, the current data from Navrongo were compared to previous data from the same area, while data from Kintampo were compared to published data from Sunyani. Kintampo and Sunyani are located in the same region (Brong Ahafo) but approximately 122 Km apart ( Figure 1) Sunyani lies in the Forest Zone whilst Kintampo lies within the Forest Savanah transition zone, however, both sites have similar agricultural practices, housing structure, and land geology, all of which have been reported to influence malaria epidemiology ( Baidjoe *et al*., 2016; Hu *et al*., 2016). Generally, a decreasing trend was observed from 2005 to 2017 in the proportions of the alleles associated with CQ resistance in both study sites except for *pfmdr1* 86 **Y** in Navrongo ( Figures 2A and B), which decreased from 2005/2006 to 2010 but appeared to plateau between 2012 and 2013. We, however, observed an increasing trend in the proportions of the pyrimethamine and sulfadoxine resistance alleles in *pfdhfr* and *pfdhps* respectively at both study sites from 2005 to 2013, with frequencies levelling off subsequently ( Figures 2C–F).

Summarized data from present study was compared to previous published data from Navrongo and Kintampo/Sunyani, Ghana, for *pfmdr1* and *pfcrt* point mutations ( Duah *et al.*, 2013) and for the antifolate resistance mutations ( Duah *et al.*, 2012). No data was available for the *pfcrt* 76T and pfmdr1 86Y in 2010 in Kintampo/Sunyani ( Figure 1A–B). Also, no data was available for Navrongo in 2016–2017 in all the analysis. Deep black represents 2005–2006, light black represents 2010, plain grey represents 2012–2013 and crossed grey represents 2016–2017.

### Analysis of *pfdhfr* and *pfdhps* haplotype combination distributions

We used clinical isolates for which all the *pfdhfr* and *pfdhps* SNP alleles of interest were successfully genotyped to survey allele combinations and determine their distribution between study periods. The prevalence of the quadruple allele combination ( **I**_51_ **R**_59_ **N**_108_/ **G**_437_) in Navrongo (2012–2013), Kintampo (2012–2013), Kintampo (2016–2017), and Accra (2016–2017) were 17/25 (68.0), 37/51 (72.6) 43/53 (81.1) and 58/71 (81.7), respectively ( Table 2). The frequency of I 51 R 59 N 108/G 437 was comparable across the study sites (P > 0.05 for all comparisons). Low prevalence (<10%) allele combinations in both *pfdhfr* and *pfdhps* were observed for the triple mutant allele combination ( **R**_59_ **N**_108_/ **G**_437_, **I**_51_ **R**_59_/ **G**_437 _and **I**_51_ **N**_108_/ **G**_437_) and these were also comparable across the study sites (P > 0.05 for all haplotypes) ( Table 2).

**Table 2.** Temporal trends of *SP drug resistance haplotypes* from 2012 to 2017 by study period.

Haplotype	Kintampo 2012-2013, n = 51 (%)	Navrongo 2012-2013, n = 25 (%)	P-value
**IRNG**	37 (72.6)	17 (68.0)	***0.789***
**RNG**	6 (11.8)	2 (8.0)	***0.714***
**IRG**	4 (7.8)	4 (16.0)	***0.427***
**ING**	4 (7.8)	2 (8.0)	***1***
	Kintampo 2012-2013, n = 51 (%)	Kintampo 2016-2017, n = 53 (%)	
**IRNG**	37 (72.6)	43 (81.1)	*0.356*
**RNG**	6 (11.8)	6 (11.3)	***1***
**IRG**	4 (7.8)	1 (1.9)	***0.201***
**ING**	4 (7.8)	3 (5.7)	***0.713***
	Kintampo 2016-2017, n = 53 (%)	Accra 2016-2017, n = 71	
**IRNG**	43 (81.1)	58 (81.7)	*1*
**RNG**	6 (11.3)	2 (2.8)	***0.0722***
**IRG**	1 (1.9)	1 (1.4)	***1***
**ING**	3 (5.7)	10 (14.1)	***0.151***

Note: Numbers include only isolates that were successfully genotyped for all the four point mutations in *pfdhfr* and *pfdhps*. Each haplotype has mutant amino acids shown in bold. **^a^**P-value based on Exact chi-square test

## Discussion

*P. falciparum* resistance to antimalarial drugs remains one of the biggest threats to the control and elimination of malaria globally. In Ghana, a change in the use of CQ to ACTs was implemented in 2005 as a result of high rate of malaria treatment failure ( Duah *et al.*, 2007). In this study, we determined the prevalence of alleles associated with CQ and antifolate resistance using clinical isolates from three malaria endemic regions with varying transmission intensities in Ghana. We observed a decreasing prevalence of CQ resistance-associated alleles but an increasing prevalence of SP resistance-associated alleles. The distribution of the alleles across the three study sites were not significant, except for *pfdhps* 437G which was significantly higher in Accra compared to Navrongo and Kintampo. The frequency of *pfdhfr/pfdhps* haplotypes in 2012–2013 and 2016–2017 were not significantly different across the three study sites. Both *in vitro* and molecular surveillance studies have associated CQ resistance mainly with the *pfcrt* 76T allele, but also with *pfmdr1* 86Y and 184F alleles. *Pfcrt* 76T *and pfmdr1* 86Y mutant alleles have also been reported to decrease *P.* falciparum ** susceptibility to amodiaquine but increase parasite sensitivity to dihydroartemisinin, lumefantrine and mefloquine ( Gresty *et al.,* 2014; Veiga *et al.,* 2016). Despite the use of ACTs (artemether-lumefantrine, artesunate-amodiaquine, and dihydroartemisinin-piperaquine) in Ghana since 2005, decreasing prevalence of *pfcrt* 76T and *pfmdr1* 86Y mutant alleles were observed in this study when compared to study by Duah and colleagues in 2013 ( Duah *et al.*, 2013). This shows a gradual decline in the frequencies of these alleles since the discontinuation of CQ as an antimalarial in Ghana, this observation is consistent with findings in other malaria endemic populations in east Africa such as Tanzania, Malawi , Kenya and Zambia where artemether lumefantrine is the first-line drug for uncomplicated malaria ( Mohammed *et al.*, 2013; Mwai *et al.*, 2009; Mwanza *et al.*, 2016). A study in Kenya posits that the K76 is preferential selection by Artemeter Lumefantrine(AL) ( Achieng *et al.*, 2015) The fitness cost of harbouring the mutant alleles is thought to select against them in favour of the non-resistant background alleles ( Babiker *et al.*, 2009; Kiarie *et al.*, 2015). Unlike *pfcrt* 76T and *pfmdr1* 86Y, the prevalence of *pfmdr1* 184F mutant allele (65%) appears to have not varied so much from 2005 to 2017 when compared to the 43% to 69% prevalence reported from 2005 to 2010 ( Duah *et al.*, 2013). Contrary to this observation, a study in Tanzania reported an increasing prevalence of *pfmdr1* N86 and 184F following the introduction of artemether-lumefantrine ( Thomsen *et al*., 2011). Notably, parasites that have a combination of *pfmdr1* mutant alleles (N86, 184F and D1246) are reported to have reduced sensitivity to artemether-lumefantrine treatment ( Baliraine & Rosenthal, 2011; Happi *et al*., 2009; Kavishe *et al*., 2014). Other studies have also linked duplication of *pfmdr1* to resistance to partner drugs of ACTs (Rodrigues, Henriques et ( Borges *et al.*, 2011; Rodrigues *et al.*, 2010).

Although high frequencies of the point mutations implicated in the development of resistance to antifolates were reported before the change in malaria treatment guidelines in 2005 in Ghana, the drug is still in use for intermittent preventive treatment of malaria in pregnancy (IPTp) and also recommended for seasonal malaria chemotherapy (SMC) among children under five in areas of high but seasonal malaria transmission. The percentages of the *pfdhfr* 51I (81%), 59R (82%), 108N (88%) and *pfdhps* 437G (88%) mutant alleles reported in this study are relatively higher when compared to the 71%, 42%, 64% and 80% prevalence reported in a recent study in a neighbouring country, Burkina Faso, using samples obtained in 2010 ( Cisse *et al.*, 2017). SP was used as a second-line treatment for uncomplicated malaria in both countries until 2005 when its usage was restricted for IPTp ( Koram *et al.*, 2005; Tahita *et al.,* 2015). In Burkina Faso, resource persons are engaged at the community level to promote IPTp uptake and referrals to antenatal clinics (ANCs) whereas IPTp in Ghana is taken at the ANCs and health care centres ( Gies *et al.*, 2009; Hill *et al.*, 2013). With the aforementioned strategies there is likely increased compliance in Burkina Faso compared to Ghana and this may explain the low resistance in the former. Furthermore, since drug resistance evolution is spatiotemporal the differences in periods of sampling could also account for the differences observed. The high prevalence may be due to SP intervention in groups such as pregnant women and young children acting as reservoirs of infections with resistance alleles as a direct consequence of continuous use of SP in IPTp and SMC campaigns that fuel transmission of these alleles in the general population. Another important factor may be the unauthorized use of SP for self-medication as it is readily available at health centres and pharmacy shops in the study areas ( Abuaku *et al.*, 2004), particularly because it is a single dose drug with very minimal to no adverse reactions. Co-trimoxazole is used in Ghana ( Fadeyi *et al.*, 2015), however, there is limited data on its usage in the three study sites. Besides the prevalence of HIV in Ghana is only 3 % ( Ghana AIDs Commission, 2017) and therefore the use of antifolate drugs such as cotrimazole for the management of opportunistic infections is not as widespread as the use of antifolate antimalarial drugs. Higher SP treatment failure has been correlated with the *pfdhfr/pfdhps* quintuple ( *pfdhfr/pfdhps* I _51_R _59_N _108_/G _437_E _540_) haplotypes ( Kublin *et al.*, 2002; Triglia *et al.*, 1997). Parasites habouring *pfdhfr/pfdhps* I _51_R _59_N _108_/G _437_ and I _51_R _59_N _108_/G _437_E _540_ haplotypes have been described as “partial” and “full” SP resistance, respectively ( Naidoo & Roper, 2013). In this study, no isolate was observed to carry the full resistance allele combination. This is consistent with other studies which show that though the variant quintuple mutant allele is almost fixed in east Africa, it is largely absent from West Africa ( Naidoo & Roper, 2013; Roper *et al.*, 2004). However, our data show an increased prevalence of parasite isolates that harbour other SP resistance haplotype in the 2012–2013 and 2016–2017 study periods. This suggests that selection by SP in our study settings is still continuing. These findings are corroborated by previous studies in Ghana (25%–69%) ( Duah *et al.*, 2012), Cameroon (47%) ( Chauvin *et al.*, 2015) and Equatorial Guinea (54%) ( Berzosa *et al.*, 2017), which suggests a high prevalence of variant quadruple mutant alleles in West to Central Africa. The *pfdhps* K540E point mutation, which is a surrogate for high level resistance to SP was found in a very low proportion of the clinical isolates (1%) in this study. This is consistent with reports in other countries in the sub-region including Mali and Burkina Faso ( Cisse *et al.*, 2017; Coulibaly *et al.*, 2014), and suggests that if selection is increased it might eventually lead to a higher level of SP resistance in West Africa.

The study indicates that CQ sensitive parasites have again become more common since the replacement of CQ with a variety of ACTs as first-line treatments of uncomplicated malaria in Ghana. This notwithstanding, our findings also show that between 5% to 14% of clinical infections may still carry CQ resistant parasites, which suggest that ACT partner drugs such as AQ that are widely used in Ghana may still be maintaining significant selection pressure on the *pfcrt* locus. In addition, the increasing prevalence of the *pfdhfr/pfdhps* partial SP resistance haplotypes could result in the fixation of these alleles within the parasite population. The continuous use of SP for IPTp and SMC may result in emergence of the “full” SP resistance haplotype and compromise the use of SP IPTp and SMC are the two significant sources of SP drug pressure on the parasite population in pregnant women and young children, respectively. Recent studies have shown that these interventions have contributed to reduction in maternal and child morbidity and mortality ( Coldiron *et al.*, 2017). Undoubtedly, these interventions are critical ( York, 2017), but could easily be undermined by rising resistance in these populations. Therefore, it is very important to closely monitor the prevalence of molecular markers of resistance associated with antifolate antimalarial drugs to guide policies on the continuous use of these drugs in Ghana and other African countries. There are probably other factors that contribute to the evolution of resistance markers as SP has been shown to be efficacious even in the face of fixation of SP resistant alleles ( Iriemenam *et al.*, 2012).

## Conclusion

This study reports an increasing prevalence of CQ sensitive clinical isolates after 12 years of CQ withdrawal at three different study sites that capture the eco-epidemiology of malaria in Ghana. The prevalence of the antifolate drug resistant alleles remain relatively high across the study sites. Besides, there is an increasing trend in the frequency of SP-resistance associated alleles at all sites. Taken together, these observations point to the need for a robust antimalarial drug discovery strategy to provide a vast array of alternatives for chemotherapy in readiness for the likelihood of future poor parasite response to the use of SP for prevention of malaria in pregnant women and for SMC in children. However, it is pre-mature to recommend the discontinuation of SP use due to the high prevalence of antifolate drug resistance alleles since the drug can be efficacious where there is fixation of these alleles.

## Data availability

The data supporting this article is available online at Open Science Framework: Dataset 1. Prevalence of chloroquine and antifolate drug resistance alleles in *Plasmodium falciparum* clinical isolates from three areas in Ghana. http://dx.doi.org/10.17605/OSF.IO/N2GZF ( Abugri *et al.*, 2018) under a CC0 1.0 Universal licence.
