# Supplementary material for: Prevalence of chloroquine and antifolate drug resistance alleles in Plasmodium falciparum clinical isolates from three areas in Ghana
Source: AAS Open Res. 2018 Dec 3;1:1. Originally published 2018 Apr 18. [Version 2] doi: 10.12688/aasopenres.12825.2 (PMC7185243; doi:10.12688/aasopenres.12825.2)
Supplement: Peer review history

# Peer review: Reviewer response for version 2

**DOI:** 10.21956/aasopenres.13998.r26676

**Published:** 2019-01-11

**Authors:** Edwin Kamau

**Affiliations:** 1 Walter Reed Army Institute of Research, Silver Spring, MD, USA

The authors have adequately responded to all my comments, the manuscript is now sufficient for approval.

I confirm that I have read this submission and believe that I have an appropriate level of expertise to confirm that it is of an acceptable scientific standard.

# Peer review: Reviewer response for version 1

**DOI:** 10.21956/aasopenres.13891.r26410

**Published:** 2018-06-01

**Authors:** Edwin Kamau

**Affiliations:** 1 Walter Reed Army Institute of Research, Silver Spring, MD, USA

Abugri *et al.* analyzed the prevalence of chloroquine (CQ) and sulphadoxine-pyrimethamine (SP) resistance in clinical isolates in Ghana. The authors used known molecular markers of resistance in three genes that modulate sensitivities to these two different antimalarial drugs; pfcrt and pfmdr1 for CQ resistance, and pfdhfr and pfdhps for SP resistance. Samples were collected from three different locations with different transmission intensities in two different time periods, 2012/2013 and 2016/2017. It is a well written manuscript but I have several comments.

 Abstract:

1. In the background, the authors state the policy was changed from CQ (as the first-line treatment?) to ACT due to CQ resistance. Was SP ever used as a first- or second-line treatment?
2. According to the methods section (in the main text), the studies were conducted in 2012/2013 and 2016/2017, NOT between 2012 and 2017 because no data was collected in 2014 and 2015.
3. In the results section, the second line which begins with “most”, needs to be improved. The authors should consider re-writing.

Introduction:

1. In the first line, Sub Saharan Africa consider writing Sub-Saharan Africa
2. In the second paragraph, the parasites have developed resistance to antimalarial monotherapies? It might be important to specify. I am assuming the authors meant Artemisinin monotherapies.
3. In the paragraph that introduces SP, consider removing the term “On the other hand” and “also” since this is the first time you have introduced this concept in the introduction.
4. In the same paragraph (on SP), references needed for the first and second lines.
5. When N (108) I (51) and R (59) is mentioned, it makes sense to put everything in chronologic order I (51) R (59) and N (108) which the authors have done to do so in the rest of the manuscript.
6.  The authors mention Duah *et al* 2007 ^1^ study which discussed the high prevalence of CQ resistance. However, they fail to mention the follow-up 2012 and 2013 studies although they mention the references later. To set the stage, I think the authors have to be upfront and mention what the follow-on studies by these authors (Duah *et al*. 2012 ^2^ and 2013[re-3]) found in Ghana.

Methods:

1. A map is needed to show the different locations where the samples were collected. It should be clear how far apart the distances are. Is Kintampo site at one district hospital or several hospitals?
2. Provide more information about the different field sites regardless of the fact that the authors have mentioned that more information is available elsewhere. The authors can be brief: what are the transmission rates, EIR etc.; what time of the year were the samples collected?

Results:

1. The first line is not important since it is stating the purpose of the study again which has already been mentioned. In addition, there is no point of repeating to mention that K76T, N86Y, Y184F associated with CQ, AQ and (not mentioned L) resistance because it is already mentioned in the introduction.
2. This is one of the major concerns of mine. First, samples were collected in two different time periods (2012/2013 in Kintampo and Navrongo; 2016/2017 in Kintampo and Accra). In a 5 year period, prevalence can change dramatically. Therefore, the frequency of distribution should be analyzed separately for the two time periods. If no differences are seen, then it should be stated as such but the analysis must be done separately.
3. This is follow-on to point #2 above. 437G was found to be higher in Accra and that’s why there was significant difference. This might because Accra samples were collected in 2016/2017 only. The authors need to breakdown the analysis per the period which the samples were collected such as comparing Navrongo 2016/2017 to Accra 2016/2017 only.
4. In malaria endemic regions, samples always contain mixed infections where both wildtype and mutant alleles are present. The authors have not mentioned anything about the presence of mixed infections and how such samples were analyzed.
5. Show Sunyani on the map so that the reader can get the sense of distance to Kintampo.
6. One of my other major concern with this study is the micro-epidemiology of malaria and the micro-heterogeneity in transmission. Malaria transmission can be highly heterogeneous over a small spatial scale not to mention dramatic change from season to season. Comparing data collected elsewhere (Sunyani vs. Kintampo) no matter the distance, and then treat it as if it is from the same location, this can be extremely problematic and misleading. The authors must make strong arguments and justify their point of view much more strongly than currently presented. See references below.
7. In the analysis of pfdhfr and pfdhps haplotypes, only Kintampo has samples collected in 2012/2013 and 2016/2017, comparing three different locations which Navrongo only has 2012/2013 samples/data and Accra has only 2016/2017 samples/data is not completely accurate (as presented 2012/2013 vs. 2016/2017). This analysis must be revisited. The difference might be just because the samples were collected in different locations; it is important this is possibility is ruled out.

Discussion:

1. The first paragraph of the discussion needs to capture and highlight the key findings of the study, consider re-writing the first paragraph of the discussion to capture the key findings.
2. The discussion needs to compare more critically similar studies that have been conducted in Ghana (Duah et al. 2012 and 2013) and West Africa. What additional information does the current study contribute?
3. Compare data with some of the most recent studies conducted in East Africa (studies cited here are not the most recent). This should be in the context of the first-line treatment ACTs in East Africa (AL) vs. first-line treatment ACTs in West Africa which might be driving the changes in molecular epidemiology of malaria parasites.
4. In page 7, the authors mention that differences in Burkina Faso and Ghana maybe due to differences IPTp uptake. This is an important line which needs references. State clearly what the differences are.
5. The authors speculate that high prevalence may be due to use of SP in IPT and SMC. What other antifolate drugs are being used in these communities such as cotrimoxazole for treatment of opportunistic infections in HIV-infected population which might be contributing to the increased resistance over time?  
6. IPTp have remained highly effective in locations where prevalence of SP resistance markers remain high and fixed in the parasite population. It is clear that from 2005 to 2017 that SP resistance markers are on the rise. The authors needs to discuss the implication of this trend in detail in the context of IPTp, are these mutations getting fixed in the population? How is monitoring of these markers going to influence policy makers if SP remains efficacious in IPTp not only in Ghana, but in other countries where prevalence of these markers remain high?

I confirm that I have read this submission and believe that I have an appropriate level of expertise to confirm that it is of an acceptable scientific standard, however I have significant reservations, as outlined above.

## References

[1] : Mutations in Plasmodium falciparum chloroquine resistance transporter and multidrug resistance genes, and treatment outcomes in Ghanaian children with uncomplicated malaria. *J Trop Pediatr*.2007;53(1) : 10.1093/tropej/fml076 27-31 10.1093/tropej/fml076 17158810

[2] : Surveillance of molecular markers of Plasmodium falciparum resistance to sulphadoxine-pyrimethamine 5 years after the change of malaria treatment policy in Ghana. *Am J Trop Med Hyg*.2012;87(6) : 10.4269/ajtmh.2012.12-0202 996-1003 10.4269/ajtmh.2012.12-0202 23045251PMC3516103

[3] : Increased pfmdr1 gene copy number and the decline in pfcrt and pfmdr1 resistance alleles in Ghanaian Plasmodium falciparum isolates after the change of anti-malarial drug treatment policy. *Malar J*.2013;12: 10.1186/1475-2875-12-377 377 10.1186/1475-2875-12-377 24172030PMC3819684

[4] : Factors associated with high heterogeneity of malaria at fine spatial scale in the Western Kenyan highlands. *Malaria Journal*.2016;15(1) : 10.1186/s12936-016-1362-y 10.1186/s12936-016-1362-y 27259286PMC4893231

[5] : The complex relationship of exposure to new Plasmodium infections and incidence of clinical malaria in Papua New Guinea. *eLife*.2017;6: 10.7554/eLife.23708 10.7554/eLife.23708 PMC560684628862132

[6] : Seasonal dynamics and microgeographical spatial heterogeneity of malaria along the China-Myanmar border. *Acta Trop*.2016;157: 10.1016/j.actatropica.2016.01.022 12-19 10.1016/j.actatropica.2016.01.022 26812008PMC4779690

[7] : Submicroscopic infection in Plasmodium falciparum-endemic populations: a systematic review and meta-analysis. *J Infect Dis*.2009;200(10) : 10.1086/644781 1509-17 10.1086/644781 19848588

[8] : Microgeography and molecular epidemiology of malaria at the Thailand-Myanmar border in the malaria pre-elimination phase. *Malar J*.2015;14: 10.1186/s12936-015-0712-5 198 10.1186/s12936-015-0712-5 25962514PMC4449518

[9] : Micro-heterogeneity of malaria transmission in the Peruvian Amazon: a baseline assessment underlying a population-based cohort study. *Malar J*.2017;16(1) : 10.1186/s12936-017-1957-y 312 10.1186/s12936-017-1957-y 28778210PMC5544973

# Peer review: Reviewer response for version 1

**DOI:** 10.21956/aasopenres.13891.r26335

**Published:** 2018-05-08

**Authors:** Deus S. Ishengoma

**Affiliations:** 1 National Institute for Medical Research, Tanga Centre, Tanga, Tanzania

**General comments:**

 The manuscript covers an important topic because of the following reasons.

1. Drug resistance has been one of the major problems that has significant impact on the WHO strategy of malaria control through case management, which entails prompt diagnosis and treatment with effective antimalarial drugs. Due to resistance to the drugs which were commonly used in the past, chloroquine (CQ) and sulphadoxine/pyrimethamine (SP), all endemic countries replaced them with artemisinin combination therapy (ACT). Thus, surveillance of molecular markers of resistance is critical to inform policy makers on the effectiveness of antimalarials currently used for treatment of malaria in the respective countries.
2. Amodiaquine (an aminoquinoline like CQ) and SP are still used in Ghana and other African countries. Whereas SP is used for intermittent preventive treatment in pregnant women (IPTp), amodiaquine-SP (which is an ACT) is used for seasonal mass chemoproprevention in the Sahel region of West Africa. Monitoring molecular markers of these drugs is very important in order to provide information on the performance of these antimalarials.

 

 The manuscript is well written but it needs some improvements by working on the following areas:

**Specific comments:**

1. *Abstract:*
  1. Under the methods, it is important to mention the genotyping method used, and how the data was managed in order guide the reader. For instance, it is mentioned that the samples were collected between 2012 and 2017 but the results are presented as a single time point, without any mention on the different time points.
  2. Results: Although it is mentioned in the methods that samples were collected between 2012 and 2017, this is not mentioned in the results and trends of the prevalence of the molecular markers are not shown.
  3. Conclusion: This is based on the comparison of the current and previous data which is not shown in the results presented. Authors should either revise the conclusion or incorporate the data referred to in the abstract.
2. *Methods:*
  1. The authors should explain why sampling involved children aged 2 – 14 years. They should also give reasons as to why venous blood was collected for this type of study which could be done using samples collected as dried blood spots on filter papers (by just finger prick). And why were the samples were depleted of leucocytes?
  2. Under data analysis, they should give reasons for pooling the results of 2002-2003 and 2016 2017 instead of analysing each year separately.
3. **Results:**
  1. Authors should give a baseline table showing the number of children sampled at each of the study sites and time points to guide the reader in terms of distribution of samples. They should also show how many samples (with percentage) were successfully genotyped in this table to give an idea of the amplification success (proportion of samples with genotypes) for each of the markers analysed. This will be helpful in interpreting the results in subsequent sections.
  2. In the introduction, it was stated that, “This study sought to ascertain the population trends in the prevalence of known drug-resistance-related point mutations in *pfcrt, pfmdr1, pfdhfr* and *pfdhps* in clinical isolates from three different malaria-endemic areas in Ghana a decade following the introduction of ACTs” and it was also mentioned in the data analysis section that the data was pooled for 2002-2003 and 2016-2017. However, the section on the prevalence of alleles of CQ and antifolates as well as table 1 don’t show any data on the prevalence of the alleles at different time points (trends). Such data should be incorporated in the table and summarized in the text as well.
  3. While in the text authors refer to “prevalence of alleles”, the heading of table 1 talks about the “frequency of SNPs”. These are two different things and should be reconciled. The authors should also explain how they dealt with samples which had mixed infections and therefore mixed alleles.  
  4. The prevalence of triple *dhfr* mutant alleles ( **I _51_R _59_N _108_**) is not shown in table 2 and the figures mentioned (53% in 2002/2003 and 79% in 2016/2017) are for the quadruple mutations of both *dhfr *( **I _51_R _59_N _108_**) and *dhps* (G _437_) while the prevalence of 43% and 73% of the quadruple mutation mentioned in the text is not shown in table 2. Since table 2 presents combined *dhfr* and *dhps* mutations, all the reported haplotypes should cover both genes because the reported number and prevalence are basically considered to have combined parasites with/without mutations in the two genes (as reported in table 2). Thus, the following statement, “Double mutant allele combinations of *pfdhfr* ( **I**_51_ **N**_108_, **R**_59_ **N**_108_ and **I**_51_ **R**_59_) had comparably low frequencies” cannot be shown in the same table (as for the triple *dhfr* mutations) without including the allele on *dhps*. The double mutation **I _51_G _437_** reported in text is not shown in table 2.
4. *Discussion:*
  1. The authors mentioned that, “The percentages of the *pfdhfr* 51I (81%), 59R (82%), 108N (88%) and *pfdhps* 437G (88%) mutant alleles reported in this study are relatively higher when compared to the 71%, 42%, 64% and 80% prevalence reported in a recent study in a neighbouring country, Burkina Faso (Cisse *et al*., 2017), this could be due to differences in the uptake of IPT _P_ in both countries.” However, they do not provide any data or evidence to show the differences in uptake of IPTp in the two countries.
  2. At the end of the second paragraph, authors state that, “Unlike *pfcrt* 76T and *pfmdr1* 86Y, the prevalence of *pfmdr1* 184F mutant allele (65%) appears to have not varied so much from 2005 to 2017 when compared to the 43% to 69% prevalence reported from 2005 to 2010”. The role of ACTs in the selection of *mdr1* mutations should be discussed, due to available evidence of increasing N86 and 184 **F** after introduction of ACTs as reported in some countries.
  3. This study covers molecular markers which are important for drugs used in both IPTp and SMC, the discussion should provide prominence on these interventions, in order to make a strong case for future surveillance and monitoring of these markers. This will provide an opportunity to monitor the impact of IPTp and SMC on the molecular markers of amodiaquine and SP resistance.

 

1. **Conclusion: **

The conclusion should be revised to align it to the objectives of the study which was stated at the end of the introduction; “This study sought to ascertain the population trends in the prevalence of known drug-resistance-related point mutations in *pfcrt, pfmdr1, pfdhfr* and *pfdhps* in clinical isolates from three different malaria-endemic areas in Ghana a decade following the introduction of ACTs”. Thus, together with a conclusion of the trends of the CQ markers, they should also summarise the trends of SP mutations before finishing with the possible implication of the findings.

1. Others comments **.**
  1. Authors should revise the document to take care of some few typos.

I confirm that I have read this submission and believe that I have an appropriate level of expertise to confirm that it is of an acceptable scientific standard.
